# Characterization of Microplastics and Adsorbed/Dissolved Polycyclic Aromatic Hydrocarbons in the Biggest River System in Saitama and Tokyo, Japan

**DOI:** 10.3390/nano14121030

**Published:** 2024-06-14

**Authors:** Qingyue Wang, Yojiro Yamada, Christian Ebere Enyoh, Weiqian Wang, Kenshi Sankoda

**Affiliations:** 1 Graduate School of Science and Engineering, Saitama University, 255 Shimo-Okubo, Sakura-ku, Saitama 338-8570, Japan; y.yamada.742@ms.saitama-u.ac.jp (Y.Y.); weiqian@mail.saitama-u.ac.jp (W.W.); 2 Department of Environmental and Civil Engineering, Toyama Prefectural University, 5180 Kurokawa, Imizu-shi 939-0398, Toyama, Japan; ksankoda@pu-toyama.ac.jp

**Keywords:** microplastics, polycyclic aromatic hydrocarbons, river pollution, adsorption, Japan

## Abstract

This study presents a comprehensive characterization of microplastics (MPs) and adsorbed/dissolved polycyclic aromatic hydrocarbons (PAHs) in the Arakawa River, the largest river system in Saitama and Tokyo, Japan. The MPs were sampled at various points along the river, revealing an average number density of 2.21 ± 1.48 pieces/m^3^, with a predominant size range of 0.5–1 mm. Polymer analysis indicated that polyethylene (PE) comprised the highest proportion of MPs (55.9%), followed by polypropylene (PP) (22.4%) and polystyrene (PS) (21.7%). Seasonal fluctuations in MPs concentration were observed, with the highest values in winter and the lowest in summer. An analysis of adsorbed PAHs revealed a median partition coefficient (Kd) value of 3.58 × 10^4^ L/kg for MPs, indicating their affinity for PAHs. Further PAHs analysis revealed that the PAHs with the highest mean values were bicyclic naphthalene, pyrene, and fluoranthene. A comparison with coastal MPs showed differences in PAH composition, with higher proportions of high-ring PAHs observed in coastal samples. The study also investigated the distribution of PAHs in the dissolved and suspended states in the river, finding that similar PAHs were distributed in both states, with the PAHs present in MPs being about 1/10,000 of those in the dissolved and suspended states. The study underscores the importance of the continued monitoring and management of MPs and associated pollutants in river ecosystems.

## 1. Introduction

Microplastics (MPs), defined as plastic particles with a size less than 5 mm, have been found in various aquatic environments including rivers [1]. Urban rivers, in particular, have been shown to have high concentrations of MPs due to the proximity of these water bodies to sources of plastic pollution such as stormwater runoff and wastewater treatment plants [1,2]. In Japan, MPs pollution has been documented in rivers and coastal waters, with a study finding that the concentration of microplastics in the Seto Inland Sea was among the highest in the world [3].

Aquatic life in urban rivers may be negatively impacted by microplastics due to physical damage and the transmission of toxic compounds that are adsorbed onto the plastic particles [4]. These polymers have the ability to penetrate the food chain and endanger human health [5]. Interestingly, microplastics have the ability to transport and absorb persistent organic pollutants (POPs) in the environment, which may increase the toxicity of the pollutants when consumed by animals [6]. POPs are especially dangerous due to their long-range mobility, bioaccumulative nature, persistence, and toxicity [7]. Numerous POPs, such as polychlorinated biphenyls, organochlorine insecticides, and polycyclic aromatic hydrocarbons (PAHs), have been found in studies to be present on the surfaces of microplastics [8,9,10].

Compounds with three or more fused benzene rings are known as polycyclic aromatic hydrocarbons (PAHs), and since they are often released into the atmosphere, they are commonly detected [11]. Because PAHs are persistent, widely dispersed, poorly biodegradable, carcinogenic and mutagenic, and have the propensity to bioaccumulate and biomagnify in food chains, they are important to the ecosystem [11]. PAHs are often colorless, white, or light-yellow solids that arise spontaneously as compound combinations [12]. Their lipophilic character makes them extremely soluble in organic solvents, but they have a low vapor pressure, high melting and boiling temperatures, and a very poor aqueous solubility (hydrophobic) [12]. The main causes of environmental PAHs are crude oil spills and incomplete fuel combustion [13]. After being released into the atmosphere, PAHs are deposited in both terrestrial and marine ecosystems via wet and dry deposition; surface runoff is responsible for transferring PAHs to the marine environment, which makes it a major PAH sink [13]. Microplastics can serve as carriers and reservoirs for these pollutants, as evidenced by the numerous studies that show the sorption of PAHs onto them in both lab and field samples [6]. Due to MPs’ ability to carry and re-emit pollutants when the surrounding water concentrations are lower than the sorbed levels, this presents environmental issues [1,6]. Furthermore, these MPs have the potential to release sorbed contaminants upon their ingestion by species, leading to varying degrees of toxicity [14].

The literature reviewed showed that significant research on MPs has been conducted over the past decade. However, most of this research has focused on the marine environment, with relatively less attention given to MPs in river environments. Rivers are reported to be the primary source of marine MPs [15], making it essential to consider the impact of river-borne MPs when addressing marine MP pollution. Previous research on MPs in rivers has primarily confirmed the existence and distribution of MPs, but there is limited knowledge about the detailed behavior of MPs, such as seasonal fluctuations and the specific pollutants adsorbed onto river MPs [16,17]. Understanding the concentration of MPs in the marine environment is crucial for assessing their effects on aquatic organisms, which can eventually affect human health. However, tracking MPs’ behavior and pollutant adsorption in the marine environment is challenging due to the time elapsed since their influx and the complexity of their behavior in such vast areas. Conversely, in river environments, the sources of MPs are more directly related to the river channels, and the processes before and after their inflow are clearer compared to the marine environment. This clarity allows for a more detailed understanding of the behavior and characteristics of MPs and the pollutants they adsorb. 

Therefore, this research investigates the distribution of microplastics (MPs) in the river environment and the contaminants adsorbed onto them, with a particular focus on polycyclic aromatic hydrocarbons (PAHs). The PAHs studied include Naphthalene (Nap), Acenaphthylene (Ac), Acenaphthene (Ace), Fluorene (Flu), Phenanthrene (Phe), Anthracene (Ant), Fluoranthene (FA), Pyrene (Py), Benz[a]anthracene (BaA), Chrysene (Chry), Benzo[b]fluoranthene (BbF), Benzo[k]fluoranthene (BkF), Benzo[a]pyrene (BaP), Indeno [1,2,3-cd]pyrene (IP), Dibenzo[a,h]anthracene (DahA), and Benzo[ghi]perylene (BP). These PAHs were selected due to their lack of emission regulation and potential for high adsorption concentrations.

To clarify the characteristics of PAH adsorption on river MPs, coastal MPs were also collected and analyzed for adsorbed PAHs. The study employed a combination of sampling, FT-IR polymer analysis, and GC/MS for quantifying and characterizing the MPs and their associated contaminants. By understanding the distribution and behavior of MPs and their adsorbed pollutants in river environments, this research provides valuable insights into the potential risks posed by MPs and PAHs to aquatic ecosystems and human health.

## 2. Experimental Method

### 2.1. Study Area

The Arakawa River is a 173 km long river located in Saitama Prefecture and Tokyo, Japan. It originates on Mount Kobushi in Saitama Prefecture and flows into Tokyo Bay. The river has a total catchment area of 2940 km^2^ and an average flow of 30 m^3^/s [18]. It is one of Tokyo’s major sources of tap water and, along with the Tone River, provides drinking water for approximately 20 million people in the Tokyo Metropolitan Area [https://www.waterworks.metro.tokyo.lg.jp/suigen/suigen_g.html (accessed on 14 July 2023)]. The Arakawa River is also a popular destination for tourists, with its widest section spanning 2537 m and featuring a range of activities such as hiking, fishing, and rafting [https://www.touristlink.com/japan/arakawa-river/overview.html (accessed on 14 July 2023)]. However, the river has also experienced flooding in the past, with the most recent incident occurring in February 2022 [19]. All this has caused the river to be contaminated by microplastics [18] and other organic pollutants [20]. 

### 2.2. Sample and Sample Collection

**Figure 1:**
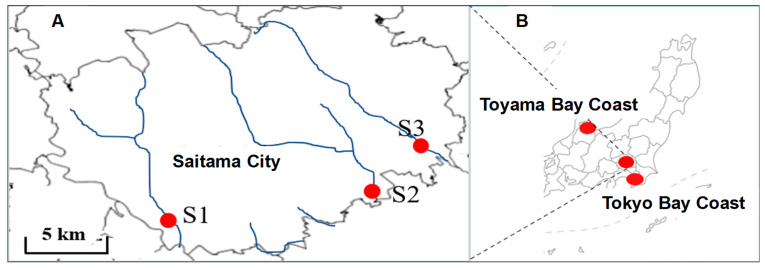
MPs collection point. (**A**) represents the sampling points of the river within the Saitama city: S1—Kamogawa/Nakabank Bridge, S2—Shibakawa/Hatchobashi, and S3—Ayase River/Nawatebashi). (**B**) Represents the sampling points within the Toyama and Tokyo Bay coasts.

Three points, i.e., S1—Kamogawa/Nakabank Bridge, S2—Shibakawa/Hatchobashi, and S3—Ayase River/Nawatebashi) (Figure 1), within the urban river were selected as the sampling points. All of these points are designated as measurement planning points in Saitama City, and a water quality survey was conducted once a month between September 2020 and March 2021. 

The MPs survey was conducted at the timing of the water quality survey. River MPs were collected using a plankton net (5502A. RIGO Co., Ltd., Tokyo, Japan) with a diameter of 45 cm, a measurement length of 180 cm, and a mesh of 0.335 mm. The plankton net was submerged in the river for 10 min and letting the river water pass through the inside of the net; drifting objects of a size exceeding the mesh of the net were caught inside the net and pulled up, and the net’s contents were washed into a brown bottle. The flow velocity, which was determined using calibrated electromagnetic current meters to represent the MPs concentrations as particles per cubic meter, was used to compute the volume of the filtered water. Following the collection, the net was washed locally before the contents were transferred to amber glass bottles and the samples were taken to the lab [18].

Next, the collection of MPs for use in the analysis of MPs-adsorbed PAHs was done 3 times, each for 10 min. In order to investigate the relationship between MPs-adsorbed PAHs and PAHs in river water, river water was collected at the same time as MPs were collected. A stainless steel bucket (3 L) was used for collecting water samples from the bridge and the samples were stored in a brown bottle.

### 2.3. Sample Pretreatment and Analysis of MPs

#### 2.3.1. Microscopic Analysis

In the lab, samples stored in the brown jar were filtered through glass fiber filters (GF/A. Whatman) with the aid of a suction pump [21]. The materials that were retained on the filters were then examined under a microscope with a digital camera (Meiji Techno Co., Ltd., Saitama, Japan). The color and size of the potential microplastics (MPs) were recorded and images were taken as needed. The MPs were classified into five size categories: 0.5–1 mm, 1–2 mm, 2–3 mm, 3–4 mm, and 4–5 mm. MPs smaller than 0.5 mm were not included in the analysis because the mesh size of the plankton net used for sampling was 0.335 mm and it was difficult to separate them with tweezers [18]. 

#### 2.3.2. Component Analysis Using FT-IR

In order to obtain a good IR spectrum, MPs were oxidized using hydrogen peroxide solution. About 5 mL of hydrogen peroxide solution was added to the centrifuge tube containing the MPs candidate, and the mixture was allowed to stand overnight. The MPs candidates inside the centrifuge tube were then transferred onto a glass fiber filter by suction filtration and stored in a petri dish for analysis of the MPs candidates by FT-IR [18]. This operation was performed only on the sample used to calculate the MPs number density, not on the MPs for adsorption PAHs analysis.

**Figure 2:**
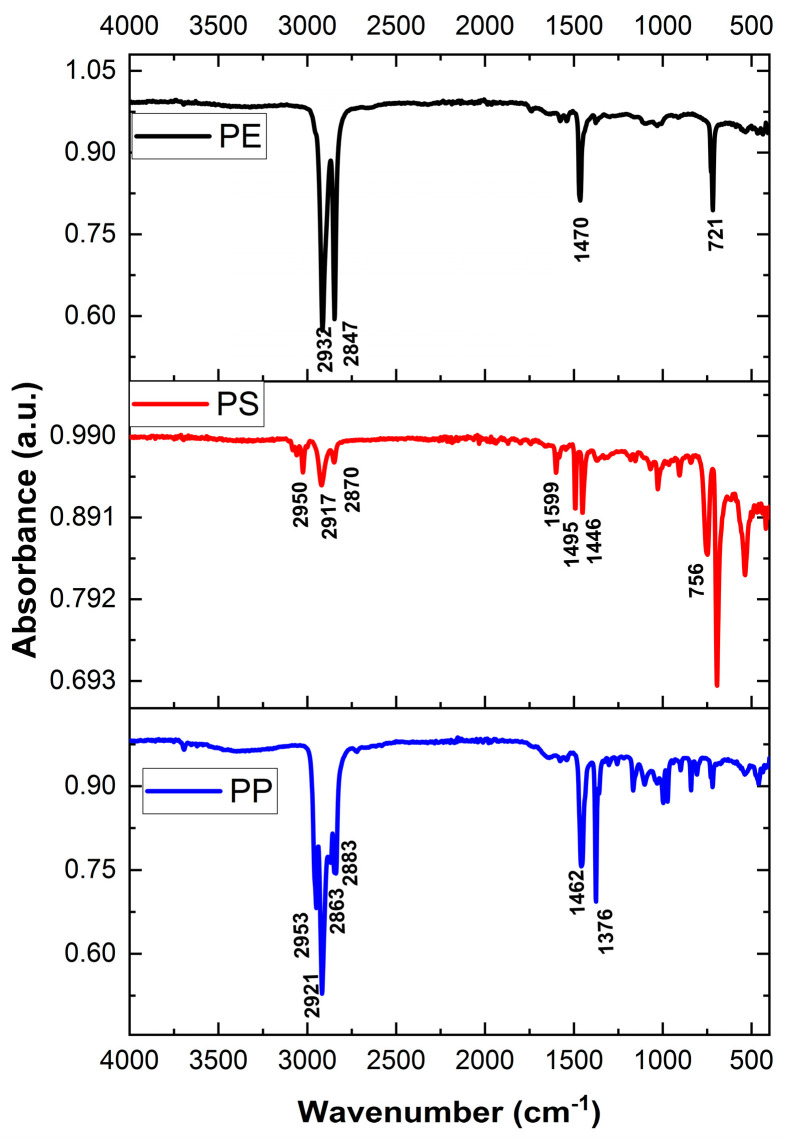
FTIR spectra for PE, PS, and PP.

The IR spectrum of the potential microplastics (MPs) was obtained using FT-IR in order to determine their polymer composition. The MPs were placed on a diamond prism, crushed, and brought into close contact with the prism before measurement. The polymer composition was identified by comparing the obtained IR spectrum with the standard spectrum of plastic. In this study, we analyzed three types of polymers: polyethylene (PE), polypropylene (PP), and polystyrene (PS) (Figure 2), which have specific densities less than 1 and are commonly found in river surface water. The standard spectrum of plastic was obtained by purchasing PE, PP, and PS plastic plates from AS ONE (Japan), measuring the IR spectrum of each using FT-IR, and incorporating it into software. The polymer was identified based on the presence of unique peaks that were similar to those of the standard spectrum in the potential MPs [21]. 

### 2.4. Extraction Method of MPs Adsorped PAHs

The method for analyzing MPs-adsorbed PAHs followed that of previous studies [13]. MPs analyzed by FT-IR were added to a 10 mL centrifuge tube. The standard amount of MPs was 50 mg, and if it was less than that, the total amount was added. Surrogate 20 µL (1 µg/L) and 10 mL of hexane were added to the centrifuge tube containing MPs. The centrifuge tube was sonicated for 10 min, the adsorbed PAHs were transferred to hexane, and hexane was removed with a Pasteur pipette. After that, hexane was added again and ultrasonic treatment was performed three times, and a total of about 30 mL of hexane was transferred to a glass container for an evaporator. This was concentrated to about 1.5 mL by an evaporator, and then purified and separated by column chromatography. Next, 4 g of silica gel was added to the beaker and was activated at 130 ° C for 3 h or more, then it was cooled in a desiccator, and then hexane was added to dissolve the silica gel. After it was poured into a column chromatograph stuffed with defatted cotton, the sample, hexane (30 mL), was poured into a hexane: dichloromethane (3:1 *v*/*v*) solution (20 mL), in that order, and 1.5mL of these eluates was re-poured into an evaporator. The sample was concentrated to 200 µL and dried with nitrogen under a gentle stream of nitrogen. It was transferred to a 2 mL brown vial using a Pasteur pipette; 1 µL of d-pyrene, which is an internal standard substance, was added, and analysis was performed by Gas Chromatography Mass spectroscopy (GC-MS: Agilent 6890 GC system with 5973 mass selective detector, Tokyo, Japan).

#### 2.4.1. Extraction of PAHs in River Water Suspension Method

The collected river water was filtered using a glass fiber filter (GF/F) that had been dried in advance and weighed. Coarse impurities such as leaves and insects in the river water sample were removed with tweezers before filtration. At this time, the equipment used for filtration and tweezers was washed with a detergent in advance and cleaned with ultrapure water. The filter used was pre-washed with acetone. The filter was dried at room temperature, finely divided with scissors, and added to a 10 mL centrifuge tube, and at the same time, surrogate 20 µL (1 µg/L) was added. After that, similar to the analysis of MPs-adsorbed PAHs, solid-phase extraction with hexane, separation and purification by column chromatography, concentration with an evaporator, and nitrogen dryness were performed, and analysis was performed with GC-MS.

#### 2.4.2. Liquid–Liquid Extraction Method

The total amount of filtered water (about 1 L) from the filtration of river water was added to a separating funnel with a content of 2 L, followed by surrogate 20 µL (1 µg/L) and hexane 200 mL. Further, a small amount of sodium chloride was added to prevent the formation of an emulsion, and the mixture was manually stirred for 10 min. After that, hexane was taken out, 200 mL of hexane was added again, and the operation of stirring was repeated. A total of 400 mL of the extract (hexane) was subjected to evaporation and nitrogen concentration, concentrated to 200 μL, and then the internal standard substance was used. A certain d-pyrene was added and the analysis was performed by GC-MS.

### 2.5. Analytical Quality Control

#### 2.5.1. Cleaning of Appliances

To prevent contamination, the glassware used was washed with detergent, acetone, and ultrapure water before use. The tweezers were used after wiping the surface with a Kimwipe soaked with acetone.

#### 2.5.2. Calibration Curve, Detection Limit, Quantification Limit

A calibration curve was prepared using a standard solution (hexane solvent) serially diluted to 6 concentrations (5 ng/mL, 10 ng/mL, 25 ng/mL, 50 ng/mL, 75 ng/mL, and 100 ng/mL). Each concentration was injected into the GC-MS in 1 µL aliquots. The calibration curve was constructed by plotting the concentration ratio of the standard substance to the internal standard substance on the horizontal axis against the peak area ratio of the internal standard substance to the standard substance on the vertical axis. This approach allowed for the correction of errors arising from device sensitivity. The resulting calibration curve demonstrated high linearity, with an R^2^ value exceeding 0.98. Subsequently, the device detection limit (IDL) and device quantification limit (IQL) were determined using the prepared calibration curve. Samples at 10 ng/mL or 25 ng/mL were analyzed seven times, and the standard deviations (σ) of their quantitative values were used to calculate IDL (3σ) and IQL (10σ). The IDL and IQL for each PAH, under each extraction method, were computed and are presented in Table 1.

#### 2.5.3. Addition Recovery Test

To validate each extraction method, an addition recovery test was conducted. In the solvent extraction method, 5 mL of solvent was added to mimic the analysis method of the sample, and 10 mg of PE pellets was added to the centrifuge tube (*n* = 2). Similarly, in the analysis method of river water suspension, the sample was dried overnight at room temperature and passed through a finely divided glass fiber filter. Then, 5 mL was added to the centrifuge tube (*n* = 2). A material mixture of PAHs standard solution (2 µg/mL) was added in both cases, with the final concentration after extraction being 100 ng/mL.

##### Solvent Extraction Method

Extraction and analysis were performed using the same method as described above. In this method, 1 L of ultrapure water was added as a substitute for river water samples in a separatory funnel (*n* = 2). A material mixture of PAHs standard solution (21 mL of 0 ng/mL) was added, ensuring that the final concentration after concentration was 100 ng/g.

##### Liquid–Liquid Extraction Method

Extraction and analysis were also performed using the same method as described above. The results of the addition recovery test for each method are summarized in Table 2.

#### 2.5.4. Operation Blank Test

An operational blank test was conducted for each extraction method. In the solvent extraction method, a 50 mg sample of PE pellets was added to a 10 mL centrifuge tube (*n* = 3). For the river water suspension, after drying overnight at room temperature, a finely divided glass fiber filter was added to a 10 mL centrifuge tube (*n* = 3). Extraction and analysis were performed using the same method as the solvent extraction method. In the liquid–liquid extraction method, 1 L of ultrapure water was added to a 2 L separatory funnel.

The results of the operation blank test for each method are summarized in Table 3. These values were utilized as the operation blank values for each extraction method. The concentration conversion was then carried out using these values by subtracting the operation blank values from the analysis results of unknown samples.

### 2.6. Statistical Analysis

Descriptive statistics were performed using Microsoft Excel 2016. Data analysis was conducted with OriginLab Pro 8 and the SPSS version 23 software package. Normality and homogeneity of variances were assessed using frequency tests and Q–Q plots. As the data met the assumptions for ANOVA, parametric tests were used. A one-way ANOVA was performed to identify any significant differences (*p* < 0.05) between the seasons, sampling points, and PAHs rings. Additionally, Pearson’s correlation analysis (CA) was conducted to evaluate potential relationships between the sampling points at a 0.05 significance level.

## 3. Results and Discussion

### 3.1. River MPs

#### 3.1.1. River MPs Concentration

After collecting and analyzing MPs from three rivers in Saitama City, a total of 960 MPs was identified. [2] reported a total MPs concentration of 804 in 29 Japanese rivers, with the Edo River having the highest concentration at 328 particles. In contrast, elsewhere, [1] found MPs abundances ranging from 440 to 1556 particles/L in surface water in Nigeria. Ref. [22] studied MPs in the surface seawaters of the Incheon/Kyeonggi Coastal Region in South Korea, reporting particle concentrations from 1602 ± 1274 to 152,688 ± 92,384 particles/m^3^. They concluded that microplastics abundance was influenced by sampling methods and spatial variations. 

The Table 4 displays the average number density of MPs, the average flow rate, and the average flow velocity at each sampling point. The MPs’ number density was calculated using the formula:

(1) Number density= Number of MPs collected/(Net area ÷ 2× river flow velocity × sampling time) 

The mean number density of MPs was 2.21 ± 1.48 Pieces/m^3^; the flow rate and velocity were 3.73 ± 0.85 m^3^/s and 0.28 ± 0.09 m/s, respectively (Table 4). The number density showed high variability, with coefficient of variation (CV) of 66.87%. This suggests that the distribution of MPs in the rivers was not uniform, with some areas potentially having significantly higher concentrations than others. Comparing the number density results with those from river MPs surveys conducted globally, our results are comparable to the results from Hongkong (7.43 item/m^3^), China [23], and Canada (1.35 item/m^3^) [24], and different to the results from Tibet (483–967 item/m^3^) [25], Indonesia (5.85 item/L) [26], and China (0.56 item/m^3^) [27]. However, the differences in target sizes and experimental methods across studies prevent a specific direct comparison.

The flow rate and the velocity of the rivers were more consistent across the sampling points, as they have moderate variations (<35%) (Table 4). The moderate variability in flow rate and velocity suggests that the rivers were relatively stable in terms of water flow during the sampling period. This stability in flow rate and velocity could influence the distribution and transport of MPs in the rivers, with higher flow rates potentially leading to greater dispersal of MPs downstream. 

Specifically, the average MPs number density peaked at sampling point S1 (Table 4). Interestingly, the average flow velocity at S1 was approximately half that of S2 and S3, suggesting that MPs entering the S1 river may have become stagnant due to the slower flow compared to the other points. This observation was supported by a significant negative correlation between MPs concentration and flow velocity (r = −0.50, *p* < 0.05). Moreover, the S1 area is surrounded by residential zones and major traffic routes, likely contributing to the higher MPs number density. 

To assess the number of MPs flowing downstream from each point, the MPs flow rate was calculated under the assumption of a uniform distribution, regardless of the water depth.

MPs flow rate = Total river flow × MPs number density(2)

The highest MPs flow rate was observed at sampling point S1, mirroring the pattern seen in the number density (Table 4). Conversely, the S3 point, which exhibited the lowest number density, also had the lowest MPs flow rate. Despite S1 having a number density five times that of S2, the difference in MPs flow rate between these points was minimal. This suggests that while MPs number density serves as an indicator of pollution levels at each point, considering the MPs flow rate is crucial for understanding the movement of MPs from rivers, especially regarding their contribution to marine environments. 

#### 3.1.2. Seasonal Fluctuations in River Microplastics

**Figure 3:**
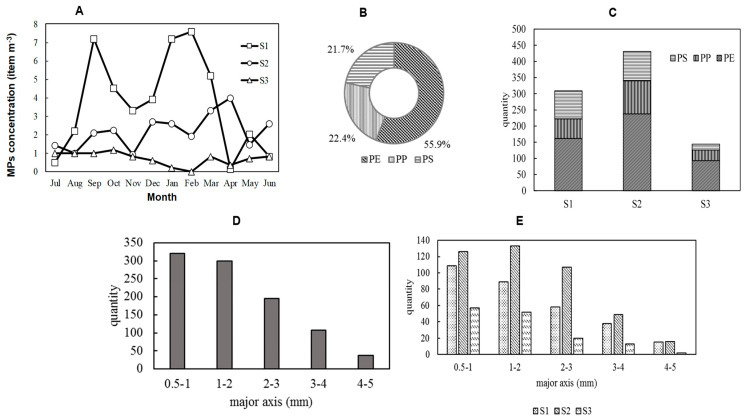
Distribution of MPs. (**A**) Seasonal variation in number density, (**B**) Polymer type composition ratio, (**C**) polymer type composition by sites, (**D**) in each major axis fraction, and (**E**) in each major axis fraction by sites.

Figure 3A illustrates the monthly variations in MPs concentration at S1, S2, and S3. At S1, the concentration ranged from 12 to 7.6 items/m^3^, exhibiting fluctuations between the maximum and minimum values. S2 showed medium monthly variations in MPs concentration. This site is likely influenced by a mix of urban runoff and natural inputs. The moderate variations at S2 can be attributed to the balance between these sources and the hydrodynamic conditions of the river, which moderate the fluctuations compared to S1 but still show some variability. In contrast, S3 showed a stable, low concentration range of 2 to 1.2 items/m^3^. Apart from S3, the lowest MPs concentrations were observed during the summer months (June–August). This seasonal trend can be attributed to variations in the river flow velocity, as well as to differences in MPs concentration among the sampling points [16,17]. The flow velocity peaked during the summer at all sampling points where MPs were collected, likely due to the increased water volume in the river caused by higher summer precipitation. This increase in flow velocity was supported by a significant positive correlation with the flow rate (r = 0.65, *p* < 0.05). Interestingly, no significant correlation was found with the river flow, suggesting that factors other than the overall river flow, such as local currents or discharge patterns, may play a more significant role in influencing MPs transport.

#### 3.1.3. Polymer Properties of River Microplastics

Polymer analysis using FT-IR identified the proportions of polyethylene (PE), polypropylene (PP), and polystyrene (PS) in the collected MPs, as illustrated in Figure 3B. PE had the highest abundance at approximately 55.9%, followed by PP at 22.4%, and PS at 21.7%. These ratios reflect the order of plastic production in Japan, where PE, PP, and styrene resins are predominant, making up 31% of total plastic production. Interestingly, despite PS production being roughly equivalent to PP production at around 5%, the number density of PS MPs was close to that of PP. This discrepancy can be attributed to the nature of the PS found in this study, which was entirely composed of foamy styrofoam. Styrofoam PS, containing about 50 times its polymer volume in air, has a low mass-to-volume ratio. Its soft and brittle structure makes it prone to fragmentation and degradation, likely resulting in a higher relative abundance of PS MPs compared to its production volume. The MPs polymer composition at the three sampling points in Saitama City’s rivers mirrored the plastic production order in Japan, yet highlighted the unique behavior of foamy PS in environmental settings. Previous research on 29 Japanese rivers [2] and East Asian seas [28] has similarly reported these polymer materials. However, ref. [29] found a broader variety of polymers in 100 μm mesh size samples from the T River in Japan, with the dominant polymers being 39.5% PE, 39.4% PP, and 10.3% PS, followed by 7.3% polyamide (PA), 2.0% polyurethane (PU), 1.0% polyvinyl chloride (PVC), and 0.6% polyethylene terephthalate (PET). This consistency suggests the widespread usage and persistence of these polymers in the environment. However, the findings by [29] highlight that smaller mesh sizes can capture a more diverse range of polymers, including PA, PU, PVC, and PET. This indicates that the standard mesh sizes used in sampling can influence the diversity of MPs detected.

Furthermore, Figure 3C shows that, at the S1 point, the abundance of PS exceeded that of PP. However, there was no significant difference in the polymer composition ratios between the sampling points, with PE consistently showing the highest values at all points. This suggests that the source of MPs is not a single point source where a specific polymer is entering the river at a specific location. Instead, MPs are likely entering the river from multiple diffuse sources, such as roads, indicating that surface pollution is contributing to MPs contamination in the rivers.

#### 3.1.4. Particle Size Characteristics of River Microplastics

Microscopic analysis of the major axis of MPs revealed the abundance of MPs in each size fraction, as shown in Figure 3D. MPs in the 0.5–1 mm size range were the most prevalent, accounting for approximately 33.4% of the total. The abundance of MPs decreased with increasing size, with MPs in the 4–5 mm range comprising only 3.85% of the total. Previous studies have indicated that MPs in the marine environment tend to become smaller over time. This result suggests that MPs in inland rivers have already undergone significant fragmentation. Figure 3E shows the abundance of MPs in each size fraction for each sampling point. Similar to the polymer composition, there was no significant difference in the size distribution of MPs across the different sites, further suggesting that river MPs are likely contributed by diffuse sources of surface pollution.

### 3.2. River MPs Adsorbed PAHs

#### 3.2.1. Quantitative Results and Component Characteristics of River MPs’ Adsorption PAHs

Table 5 presents the quantitative results of MPs-adsorbed PAHs for sampling points S1 and S2, not detected in S3. The average values were calculated assuming that undetected samples had a concentration of 0, which may place them below the detection limit. The number of detections for each PAH is also shown in Table 5. The total concentration of PAHs (∑PAHs) was 488 ± 478 ng/g at S1 and 304 ± 157 ng/g at S2. The concentrations were comparable to those reported in other studies conducted elsewhere such as in sea water in China (13.8–888 ng/g [30] and (282.4–427.3 ng/g [31]), and Indonesia (19.19–408 ng/g) [8], while lower compared to concentrations reported for sea and lake water in Japan (1730–27,100 ng/g) [32] and Italy (3400–119,000 ng/g) [33]. However, these studies have reported different sizes of MPs; therefore, it is not possible to make a direct comparison. It was generally observed that smaller MPs tended to have a higher concentration of PAHs.

**Figure 4:**
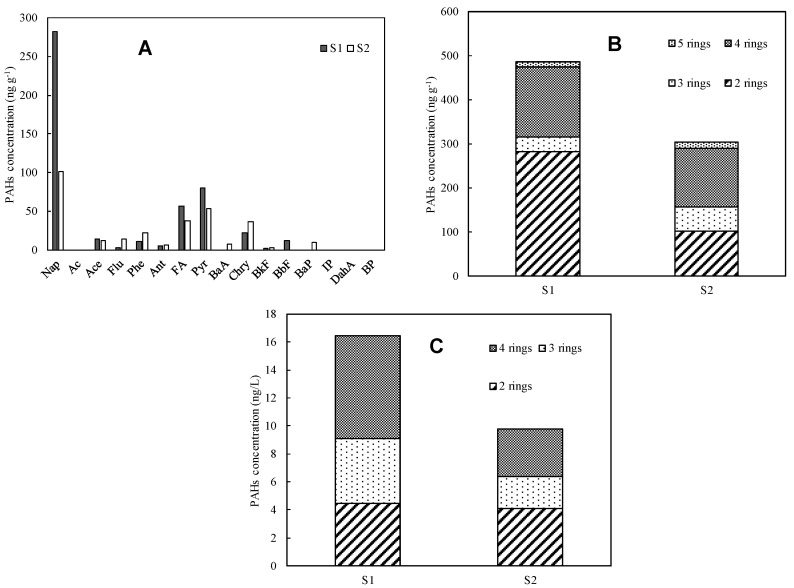
MPs-adsorbed PAHs. (**A**) Average concentration, (**B**) components, and (**C**) dissolved PAHs components.

Figure 4A shows the average concentration of each PAH adsorbed on MPs at each sampling site. The average PAH concentrations displayed similar trends at both sites where MPs were collected. Among the 16 PAHs analyzed, naphthalene, a bicyclic PAH, had the highest mean values (S1: 57.7%, S2: 33.2%). This was followed by pyrene (S1: 16.4%, S2: 17.5%) and fluoranthene (S1: 11.6%, S2: 12.3%). Certain PAHs, including six-ring PAHs (indeno[1,2,3-cd]pyrene, benzo[ghi]perylene), some five-ring PAHs (dibenz[a,h]anthracene), and a two-ring PAH (acenaphthylene), were not detected at these points.

The composition of MPs-adsorbed PAHs, categorized by the number of rings, is shown in Figure 4B. The majority were 2-ring PAHs (S1: 57.7%, S2: 33.2%) and 4-ring PAHs (S1: 32.7%, S2: 44.2%). In contrast, the proportion of 3-ring and 5-ring PAHs was much smaller.

To investigate the component characteristics of MPs-adsorbed PAHs, we analyzed the correlation between the octanol–water partition coefficient (log Kow) and the concentration of each PAH. The analysis revealed a significantly negative correlation (r = −0.53, *p* < 0.05). Previous studies have reported that the affinity between organic compounds and MPs increases with higher log Kow values [27], indicating a stronger affinity with MPs. However, the results of this study are inconsistent with those findings. One possible reason for this discrepancy is that the water environment surrounding MPs is more inclined towards low-ring PAHs. Table 6 presents the results of PAHs analysis in water-soluble rivers, and Figure 4C shows the ratio of PAHs in the dissolved state by the number of rings. Low-ring (two and three rings) PAHs accounted for 58.4%, indicating their dominance in the dissolved state. This suggests that low-ring PAHs are more prevalent in the water environment, influencing their adsorption characteristics on MPs. Moreover, the behavior of PAHs in aquatic environments can be affected by various factors such as water temperature, flow rate, and the presence of other organic materials, which might have influenced the observed adsorption patterns [6]. The lower-ring PAHs, which are more soluble in water, may have higher mobility [34] and, therefore, a greater likelihood of coming into contact with MPs, leading to their increased adsorption despite their lower log Kow values. This highlights the importance of considering the specific conditions of the study area when interpreting the affinity of PAHs for MPs [35].

##### Isomeric Ratios

To estimate the origin of PAHs detected in MPs, we analyzed the component ratios (Diagnostic Ratios: DRs) of PAH isomers [13]. For example, an anthracene—(phenanthrene + anthracene) ratio greater than 0.1 indicates a thermal origin, while a ratio less than 0.1 indicates a petroleum origin. Similarly, a fluoranthene—(fluoranthene + pyrene) ratio greater than 0.4 suggests a thermal origin, and a ratio less than 0.4 indicates a petroleum origin [13,36].

The average DRs values of MPs-adsorbed PAHs in this study were 0.31 at the S1 point and 0.24 at the S2 point for the anthracene—(phenanthrene + anthracene) ratio, suggesting mixed origins. For the fluoranthene—(fluoranthene + pyrene) ratio, the values were 0.41 at both S1 and S2, indicating a thermal origin. Additionally, the fluoranthene—(fluoranthene + pyrene) ratios in the water-soluble state and the suspended state in the river were 0.43 and 0.71, respectively. These values are similar to those found in MPs, suggesting that the PAHs in MPs may have originated from the surrounding water sources.

#### 3.2.2. Seasonal Variation of River MPs’ Adsorbed PAHs

**Figure 5:**
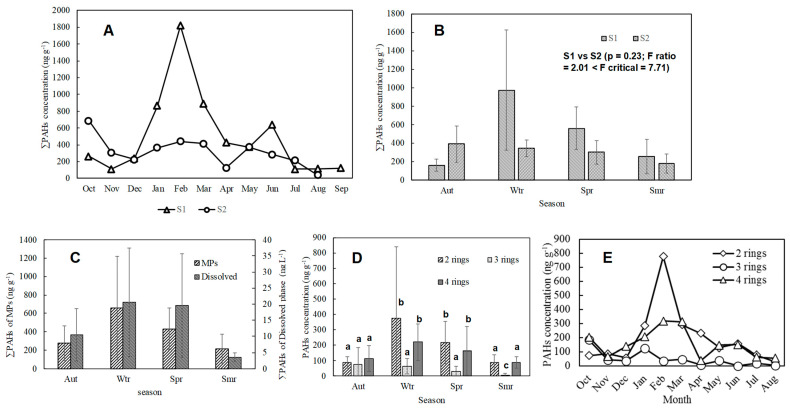
MPs fluctuations in adsorbed PAH by (**A**) month and (**B**) season. (**C**) Comparison of seasonal ∑PAHs and dissolved ∑PAHs and ring composition by (**D**) season and (**E**) month. Bars with similar alphabets are not statistically significantly different (*p* > 0.05).

Figure 5A shows the monthly variations in PAHs adsorbed on MPs in the river. At the S1 point, the highest concentration of PAHs adsorption was recorded in February (1820 ng/g), while the lowest concentration was observed in November (113 ng/g). Conversely, at the S2 point, the maximum concentration was observed in October (670 ng/g), and the minimum in April (125 ng/g). The months showing the maximum and minimum concentrations of PAHs adsorption did not match between the two sites.

Figure 5B shows the seasonal variations in PAHs adsorbed on MPs in the river. The seasons are classified as autumn (September–November), winter (December–February), spring (March–May), and summer (June–August). The mean distribution between PAHs adsorbed at different seasons showed no significant differences (*p* = 0.23; F ratio = 2.01 < F critical = 7.71). However, at the S1 point, the highest concentration of adsorbed PAHs was in winter (973 ± 651 ng/g), followed by spring (562 ± 232 ng/g) and summer (256 ± 185 ng/g), and the lowest was in autumn (163 ± 63.4 ng/g). Conversely, at the S2 point, the highest concentration was in autumn (391 ± 197 ng/g), followed by winter (344 ± 90.4 ng/g) and spring (302 ± 127 ng/g), and the lowest was in summer (179 ± 103 ng/g). The results indicate that the amount of PAHs adsorbed on MPs tends to be highest in winter at both S1 and S2 points. In contrast, the lowest concentrations were observed in summer at both points. This trend suggests that the amount of PAHs adsorbed on MPs increases during the winter compared to the summer.

A correlation analysis was conducted between the average temperature on the MPs collection day and the PAHs concentration, revealing a significant negative correlation (r = −0.49, *p* < 0.05). This suggests that lower temperatures may contribute to the observed increase in MPs adsorbed PAHs during the winter months. The relationship between PAHs adsorption on MPs and temperature can be explained by considering the heat of adsorption and the photolysis of PAHs in the atmosphere and water. First, the heat of adsorption for MPs and PAHs is positive, indicating that lower water temperatures result in increased adsorption due to equilibrium shifts. Additionally, atmospheric PAHs, which deposit into the water, are influenced by seasonal changes in temperature [37]. During the summer, higher temperatures and increased solar radiation enhance the photolysis of atmospheric PAHs, leading to their reduction. Consequently, this decrease in atmospheric PAHs in summer results in lower PAHs concentrations in the water and, subsequently, on MPs. Moreover, fossil fuel combustion, a primary source of PAHs, typically increases in winter due to heating needs, contributing to higher atmospheric PAHs levels in winter. Seasonal studies have shown that atmospheric PAHs decrease in summer, correlating with the observed lower PAHs levels in water during this period. Photodegradation of PAHs occurs not only in the atmosphere but also in water. Since MPs in surface water are exposed to significant light energy, PAHs adsorbed on MPs are likely subjected to photodegradation. Although MPs adsorption slows the decomposition rate, it still occurs, and the half-life of PAHs on MPs is longer than it is in water, yet they undergo photodecomposition [37]. While no significant correlation was found between the concentration of dissolved PAHs and MPs, a seasonal trend was observed. Both the MPs adsorption PAHs concentration and the dissolved PAHs concentration showed higher levels in winter, spring, and autumn, and decreased levels in summer (Figure 5C). This seasonal pattern further supports the influence of temperature and photolysis on PAHs dynamics in the environment. Generally, the adsorption of PAHs by MPs is driven by several mechanisms [6,14]. Hydrophobic interactions play a significant role, as both PAHs and MPs are hydrophobic, leading to mutual attraction and adsorption. Van der Waals forces, which are weak intermolecular forces, facilitate the adherence of PAHs to MP surfaces. Additionally, π-π interactions occur between the aromatic rings in PAHs and MPs, enhancing adsorption. The surface area and the porosity of MPs are also crucial, as higher surface areas and porous structures provide more sites for adsorption. Surface functional groups on MPs can form hydrogen bonds or electrostatic interactions with PAHs, further aiding the adsorption process. Diffusion mechanisms allow PAHs to move across MP surfaces or into internal pores. Environmental factors such as temperature, pH, salinity, and the presence of other compounds can influence the adsorption rates and capacities. These mechanisms collectively explain how MPs act as vectors for PAHs, transporting them through aquatic environments and potentially introducing them into the food chain, thereby exacerbating their environmental impact [6].

Analyzing seasonal fluctuations revealed that two-ring and four-ring PAHs exhibited maximum values in winter and minimum values in summer, similar to the trend observed for ∑PAHs (Figure 5D). ANOVA showed that by seasons, there was no significant differences (*p* = 0.72). However, specific rings, i.e., three rings during summer and two and four rings during winter and spring seasons showed significant differences (*p* < 0.05). Interestingly, while the concentration of PAHs in autumn (75.5 ± 109 ng/g) was higher than that in winter (64.6 ± 48.7 ng/g), the lowest value for three-ring PAHs was observed in summer (6.65 ± 12.3 ng/g), similar to the trend seen in two-ring and four-ring PAHs (Figure 5D). However, monthly trends of PAHs (Figure 5E) showed the highest distribution in February for two and four rings, and in January for three rings. The difference in concentration between the maximum and minimum values was smallest for four-ring PAHs (2.5-fold), followed by two-ring PAHs (4.3-fold), and largest for 3-ring PAHs (11-fold). The increasing stability of PAHs with a higher number of rings likely contributed to the lower impact of photodegradation on PAHs with more rings, even during summer when photodegradation is most pronounced for PAHs with fewer rings [37].

#### 3.2.3. Underwater PAHs Distribution

To investigate the distribution of PAHs between MPs and river water, the partition coefficient (Kd value) was calculated by dividing the concentration of ΣPAHs in MPs by the concentration of ΣPAHs in the dissolved state. The maximum Kd value for MPs compared to the dissolved state was 8.68 × 10^5^ L/kg (observed in February at S1), with a median value of 3.58 × 10^4^ L/kg. PAHs were also detected in suspended matter in rivers (Table 7), and the Kd value was calculated in the same manner as it was for MPs, by dividing the concentration of suspended ΣPAHs by the concentration of dissolved ΣPAHs.

The maximum Kd value for suspended matter compared to the dissolved state was 3.15 × 10^5^ L/kg, with a median value of 3.65 × 10^4^ L/kg. The MPs analyzed in this study have a size of 5 mm, while suspended matter is filtered to particles smaller than 0.7 µm. These minute particles have a much larger specific surface area compared to MPs. Previous studies have shown that the adsorption capacity increases with specific surface area [38]. Therefore, despite the difference in particle size, the MPs in this study exhibited a similar amount of PAHs adsorption as the suspended matter, indicating that MPs have a higher affinity for PAHs than suspended matter. In other studies, Lee [39] determined partition coefficients between PE MPs and seawater for eight PAHs, including phenanthrene and fluoranthene. The partition coefficients for phenanthrene and fluoranthene were found to be high, indicating that MPs have a significant sorption capacity for these compounds. All categories of POPs are hydrophobic (water-repellent) and lipophilic (fat-attracting) compounds. In aquatic environments, they tend to bind strongly to solid particles and avoid the aqueous phase, leading to their persistence.

##### Comparing the Masses of PAHs Dissolved, Particulate, and Adsorbed MPs in River Water

To compare the masses of dissolved, *particulate/suspended*, and MPs in river water (per cubic meter, m^3^), the following calculations were made. It was assumed that the mass of 1 L (L) of water in the dissolved state is 1 kg (kg). For the mass of suspended matter, the mass difference before and after drying of the filter used during the analysis of suspended-state PAHs was used. The formula used to calculate the abundance of MPs is as follows:

Mass of MPs contained in 1 m^3^(g/m^3^) = MPs number density (item m^−3^) × Average MPs mass (g item^−1^) (3)

**Figure 6:**
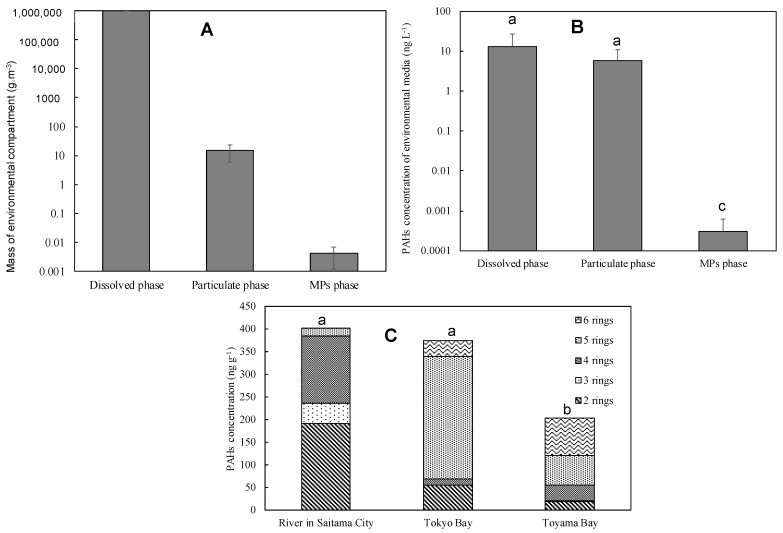
(**A**) Mass of each phase contained in water 1 m^3^; (**B**) ∑PAHs concentration present as each phase contained in 1 L water and (**C**) abundance of MPs-adsorbed PAHs in rivers and coasts by number of rings. Bars with similar alphabets are not statistically significantly different (*p* >0.05).

Here, the average MPs mass was calculated using the total MPs weight used in the analysis of river MPs (1.01 g) divided by the total number of MPs used in the analysis (724 items), resulting in 1.40 × 10^−3^ g/item. The average values for dissolved, suspended, and MPs masses for all samples are shown in Figure 6A.

When comparing the abundance of two solid phases (suspended state and MPs), the suspended state (14.9 ± 9.01) shows an abundance about 1000 times larger than MPs (4.13 × 10^−3^ ± 2.90 × 10^−3^), indicating that the mass of MPs in river water is very small compared to the suspended state.

Next, to investigate the distribution of PAHs in water among the dissolved, suspended, and MPs phases, the ΣPAHs abundance was calculated for each phase per liter of river water. The following formulas were used for the calculations:

(4) $$\sum \mathrm{PAHs}\;\mathrm{that}\;\mathrm{exist}\;\mathrm{as}\;\mathrm{MPs}\;\mathrm{per}\;\mathrm{liter}\;\mathrm{of}\;\mathrm{water}\left(\mathrm{ng}/L\right)=\mathrm{Water}\;1\;m^{3}\;\mathrm{Mass}\;\mathrm{of}\;\mathrm{MPs}\;\mathrm{contained}\;\mathrm{in}\;\mathrm{the}\;\mathrm{hit}\left(g/m^{3}\right)\times \sum \mathrm{PAHs}\;\mathrm{concentration}\left(\mathrm{ng}/g\right)\times 10^{-3}$$

(5) $$1\;L\;\mathrm{Water}\;\mathrm{PAHs}\;\mathrm{that}\;\mathrm{exist}\;\mathrm{as}\;\mathrm{suspensions}\;\mathrm{contained}\;\mathrm{in}\;\mathrm{the}\;\mathrm{hit}\;=\;\frac{\mathrm{Analytical}\;\mathrm{values}\;\mathrm{from}\;\mathrm{GC}/\mathrm{MS}(\mathrm{ng}/\mathrm{mL})\;-\;\mathrm{Blank}\;\mathrm{values}\;(\mathrm{ng}/\mathrm{mL})}{\mathrm{Surrogate}\;\mathrm{recovery}\;\mathrm{rate}}\times \frac{\mathrm{Final}\;\mathrm{liquid}\;\mathrm{volume}\;(200\;\mathrm{µL})}{\mathrm{Amount}\;\mathrm{of}\;\mathrm{water}\;\mathrm{used}\;\mathrm{in}\;\mathrm{the}\;\mathrm{analysis}(L)}$$

The ΣPAHs, which exist as suspensions contained in 1 L of water, are calculated by summing all PAHs. The results are shown in Figure 6B. Similar amounts of PAHs were distributed in the dissolved and suspended states, while the PAHs present in the MPs phase were about 1/10,000 of them. The amounts in the dissolved and particulate/suspended phase showed no significant difference (*p* > 0.05) but were significantly different from the MPs phase (*p* < 0.05). This suggests that MPs are of low importance in the transport of chemicals in rivers. However, with the increasing production volume of plastics, the role of MPs as a medium for transporting chemical substances may change in the future.

#### 3.2.4. Comparison of River and Coastal Microplastic Adsorption PAHs

To elucidate the adsorption characteristics of PAHs on river MPs, MPs collected from the coasts of Toyama Bay and Tokyo Bay were analyzed and compared with river MPs. Figure 6C shows the adsorption PAHs of river MPs in Toyama Bay and Tokyo Bay in Saitama City categorized by ring number. The analysis of MPs drifting on the coasts of Tokyo Bay and Toyama Bay revealed the presence of ∑PAHs similar to those found in river MPs. The mean river MPs adsorbed PAHs at the Saitama City and Toyama Bay coasts showed no significant differences (*p* > 0.05) but they were significantly different from those at the Tokyo Bay coast (*p* < 0.05). However, while the proportion of five–six-ring PAHs was 4.4% in river MPs, it accounted for 78.4% in coastal drift MPs, indicating that high ring PAHs were dominant in coastal drift MPs. Coastal drift MPs travel longer distances compared to river MPs, and their routes may include areas with high levels of PAH pollution. Additionally, polycyclic PAHs have a strong affinity for MPs and are not easily decomposed, leading to their prolonged retention on MPs even after the surrounding PAH concentration decreases. Therefore, there appears to be a difference in the composition of adsorbed PAHs between rivers and coasts due to the transport routes of MPs and the physical characteristics of PAHs.

## 4. Conclusions and Recommendation

This study investigated the characteristics and distribution of microplastics (MPs) and polycyclic aromatic hydrocarbons (PAHs) in the biggest rivers of Saitama and Tokyo, Japan. The analysis revealed that MPs are primarily composed of polyethylene (PE), polypropylene (PP), and polystyrene (PS), consistent with previous studies. Seasonal fluctuations showed that the adsorption of PAHs onto MPs was highest in winter and lowest in summer, with significant negative correlations between PAH concentration and average temperature. Furthermore, the PAHs were distributed among the dissolved, suspended, and MP phases, with MPs contributing a smaller fraction compared to the other phases. The study also highlighted differences in PAH composition between riverine and coastal MPs, suggesting that MPs’ transport routes and physical characteristics of PAHs play a crucial role. Based on the results, it is recommended to conduct further studies to investigate the sources and transport pathways of MPs and PAHs in river systems. Long-term monitoring programs can help assess trends and seasonal variations, contributing to effective management strategies. Additionally, efforts to reduce plastic waste and improve waste management practices should be intensified to minimize the input of MPs into rivers and oceans. Public awareness campaigns can also play a crucial role in reducing plastic pollution and its associated impacts on aquatic ecosystems.

## Figures and Tables

**Table 1:** Detection limit and quantification limit.

PAHs	IDL (ng/mL)	IQL (ng/mL)	Solvent Extraction Method		Liquid–Liquid Extraction Method	
			LOD (ng/g)	LOQ (ng/g)	LOD (ng/L)	LOQ (ng/L)
Nap	0.64	2.12	2.55	8.49	0.13	0.42
Ac	0.45	1.5	1.8	6	0.09	0.3
Ace	0.28	0.95	1.14	3.79	0.06	0.19
Flu	0.31	1.02	1.23	4.08	0.06	0.2
Phe	0.37	1.23	1.48	4.93	0.07	0.25
Ant	1.09	3.63	4.36	14.53	0.22	0.73
FA	0.67	2.23	2.68	8.94	0.13	0.45
Pyr	0.57	1.92	2.3	7.66	0.11	0.38
BaA	1.53	5.11	6.13	20.45	0.31	1.02
Chry	1.23	4.09	4.91	16.36	0.25	0.82
BkF	1.04	3.48	4.17	13.91	0.21	0.7
BbF	2.32	7.72	9.26	30.88	0.46	1.54
BaP	4.17	13.9	16.69	55.62	0.83	2.78
IP	2.27	7.56	9.07	30.25	0.45	1.51
DahA	7.14	23.79	28.55	95.17	1.43	4.76
BP	3.72	12.42	14.9	49.66	0.74	2.48

**Table 2:** Addition recovery test results.

PAHs	Solvent Extraction Method: MPs (*n* = 2)		Solvent Extraction Method: Suspension (*n* = 2)		Liquid–Liquid Extraction Method (*n* = 2)	
	Recovery Rate (%)	% RSD	Recovery Rate (%)	% RSD	Recovery Rate (%)	% RSD
Nap	73.0	18.1	60.0	10.2	77.2	0.6
Ac	59.4	15.2	51.8	9.6	69.0	1.2
Ace	66.6	14.9	30.2	3.2	35.5	0.9.
Flu	75.3.	10.4	45.0	9.7	57.2	1.3
Phe	62.3	12.9	71.1	12.2	118.9	10.4
Ant	76.9	13.4	77.1	10.1.	112.1	0.5
FA	72.3	11.1	81.5	6.7	125.1	6.2
Py	86.9	14.6	86.0	7.3	144.5	5.5
BaA	54.4	4.9	128.5	0.2	110.7	5.5
Chry	61.0	9.7	149.0	1.2	132.6	2.2
BbF	30.1	6.0	102.2	2.0	98.5	1.7
BkF	47.3	8.2	116.0	9.8	126.0	5.4
BaP	38.1	14.5	117.6	9.0	105.3	5.6
IP	32.3	2.7	121.0	2.2	107.0	1.6
DahA	31.1	32.2	120.2	3.2	106.8	0.2
BP	36.2	5.3	86.8	4.2	104.4	2.8
d-Chry	51.1	1.6	89.6	29.3	72.3	0.1
d-Ace	59.9	18.6	45.5	29.2	37.6	5.0
d-Nap	54.0	14.4	42.4	29.9	29.9	5.2.
d-peryrene	39.5	1.6	72.5	33.5	64.4	2.3
d-Phe	82.3	18.7	58.7	25.6	63.3	10.2

**Table 3:** Operation blank test result (ng/mL).

PAHs	Solvent Extraction Method: MPs (*n* = 3)			Solvent Extraction Method: Suspension (*n* = 3)			Liquid–Liquid Extraction Method (*n* = 3)		
	Average	Standard Deviation	% RSD	Average	Standard Deviation	% RSD	Average	Standard Deviation	% RSD
Nap	2.24	0.17	7.64	7.33	6.50	88.57	0.66	0.51	78.05
Ac	――――	――――	――――	――――	――――	――――	――――	――――	――――
Ace	――――	――――	――――	――――	――――	――――	――――	――――	――――
Flu	――――	――――	――――	――――	――――	――――	――――	――――	――――
Phe	6.55	1.03	15.65	5.77	1.93	33.47	6.09	1.41	23.14
Ant	2.05	2.05	100.0	――――	――――	――――	――――	――――	――――
FA	3.33	0.27	8.04	1.48	1.48	100.0	――――	――――	――――
Py	――――	――――	――――	――――	――――	――――	――――	――――	――――
BaA	3.76	0.68	17.96	7.23	0.94	12.96	――――	――――	――――
Chry	――――	――――	――――	――――	――――	――――	――――	――――	――――
BbF	――――	――――	――――	――――	――――	――――	――――	――――	――――
BkF	――――	――――	――――	――――	――――	――――	――――	――――	――――
BaP	――――	――――	――――	――――	――――	――――	――――	――――	――――
IP	――――	――――	――――	――――	――――	――――	――――	――――	――――
DahA	――――	――――	――――	――――	――――	――――	――――	――――	――――
BP	――――	――――	――――	――――	――――	――――	――――	――――	――――

――――: not conducted.

**Table 4:** Average number density and flow rate of MPs, average river flow rate, and velocity at each point.

	S1	S2	S3	Mean ± SDV	CV (%)
Number density (Pieces/m^3^)	3.7	2.2	0.74	2.21 ± 1.48	66.87
Flow rate (m^3^/s)	3.1	4.7	3.4	3.73 ± 0.85	22.78
Flow velocity (m/s)	0.18	0.35	0.32	0.28 ± 0.09	32.03
MPs flow rate (Pieces/s)	10.8	9.21	2.87	7.63 ± 4.20	55.01

**Table 5:** MPs’ adsorption PAHs quantification results (ng/g).

		S1			S2		S3
PAHs	Average	Range	Number of Detections	Average	Range	Number of Detections	
Nap	28.282	45.3–1387	12/12	101	1.47–264	12/12	ND
Ac	ND	ND–ND	0/12	ND	ND–ND	0/12	ND
Ace	14.5	ND–92.3	3/12	12.4	ND-51.0	6/12	ND
Flu	2.89	ND–18.8	2/12	14.6	ND-48.5	6/12	ND
Phe	11.0	ND–75.5	5/12	21.7	ND–166	7/12	ND
Ant	4.95	ND–36.4	3/12	6.72	ND-46.9	6/12	ND
FA	56.8	ND–240	10/12	37.5	12.7–108	12/12	ND
Pyr	ND	ND–432	9/12	53.3	4.60–105	12/12	ND
BaA	ND	ND–ND	0/12	7.64	ND–33.3	4/12	ND
Chry	ND	ND–93.5	4/12	36.0	ND–142	7/12	ND
BkF	22.6	ND–17.4	1/12	3.33	ND–40.0	1/12	ND
BbF	1.45	ND–146	1/12	ND	ND–ND	0/12	ND
BaP	12.2	ND–ND	0/12	9.86	ND–118	1/12	ND
IP	ND	ND–ND	0/12	ND	ND–ND	0/12	ND
DahA	ND	ND–ND	0/12	ND	ND–ND	0/12	ND
BP	ND	ND–ND	0/12	ND	ND–ND	0/12	ND
∑16PAHs	488	113–1820	12/12	304	40.0–670	12/12	ND

ND: Not detected/Below detection limits.

**Table 6:** Quantitative results of dissolved PAHs (ng/L).

		S1			S2		S3
PAHs	Average	Range	Number of Detections	Average	Range	Number of Detections	
Nap	4.45	ND–20.3	11/12	4.07	ND–18.2	11/12	ND
Ac	0.285	ND–1.28	3/12	0.468	ND–3.09	3/12	ND
Ace	ND	ND–ND	0/12	0.0803	ND–0.59	2/12	ND
Flu	0.0361	ND–0.433	1/12	ND	ND–ND	0/12	ND
Phe	4.34	ND–18.0	11/12	1.76	ND-9.18	9/12	ND
Ant	ND	ND–ND	0/12	ND	ND–ND	0/12	ND
FA	1.55	ND–11.3	6/12	0.738	ND–3.25	6/12	ND
Pyr	5.60	ND–27.0	11/12	2.61	ND-10.5	11/12	ND
BaA	ND	ND–ND	0/12	ND	ND–ND	0/12	ND
Chry	0.183	ND–1.32	2/12	0.0653	ND–0.794	1/12	ND
BkF	ND	ND–ND	0/12	ND	ND–ND	0/12	ND
BbF	ND	ND–ND	0/12	ND	ND–ND	0/12	ND
BaP	ND	ND–ND	0/12	ND	ND–ND	0/12	ND
IP	ND	ND–ND	0/12	ND	ND–ND	0/12	ND
DahA	ND	ND–ND	0/12	ND	ND–ND	0/12	ND
BP	ND	ND–ND	0/12	ND	ND–ND	0/12	ND
∑16PAHs	16.5	2.09–53.7	12/12	9.79	2.25–21.8	12/12	ND

ND: Not detected.

**Table 7:** Suspended PAHs quantification results(ng/g).

		S1			S2	
PAHs	Average	Range	Number of Detections	Average	Range	Number of Detections
Nap	217	ND–956	7/12	280	ND–1251	10/12
Ac	0.89	ND–10.7	1/12	15.0	ND–180	1/12
Ace	2.38	ND–28.6	1/12	30.8	ND–233	3/12
Flu	ND	ND–ND	0/12	24.6	ND–268	2/12
Phe	43.2	ND–141	4/12	54.3	ND–332	4/12
Ant	ND	ND–ND	0/12	ND	ND–ND	0/12
FA	8.07	ND–46.9	4/12	21.7	ND–112	6/12
Pyr	ND	ND–ND	0/12	11.6	ND–ND	2/12
BaA	8.51	ND–ND	1/12	2.74	ND–127	1/12
Chry	12.4	ND–102	3/12	29.4	ND–32.9	3/12
BkF	9.10	ND–101	2/12	5.53	ND–163	1/12
BbF	23.8	ND–85.1	2/12	13.2	ND–66.4	1/12
BaP	58.8	ND–146	2/12	13.9	ND–158	1/12
IP	ND	ND–502	0/12	ND	ND–167	0/12
DahA	ND	ND–ND	0/12	ND	ND–ND	0/12
BP	ND	ND–ND	0/12	ND	ND–ND	0/12
∑16PAHs	377	16.4–956	12/12	502	18.3–1464	12/12

ND: Not detected.

## Data Availability

Data is provided in the manuscript.
